# Green Waste-Derived Substances Immobilized on SBA-15 Silica: Surface Properties, Adsorbing and Photosensitizing Activities towards Organic and Inorganic Substrates

**DOI:** 10.3390/nano9020162

**Published:** 2019-01-29

**Authors:** Maria Laura Tummino, Maria Luisa Testa, Mery Malandrino, Roberta Gamberini, Alessandra Bianco Prevot, Giuliana Magnacca, Enzo Laurenti

**Affiliations:** 1 Dipartimento di Chimica, Università di Torino, via P. Giuria 7, I-10125 Torino, Italy; marialaura.tummino@unito.it (M.L.T.); mery.malandrino@unito.it (M.M.); alessandra.biancoprevot@unito.it (A.B.P.); giuliana.magnacca@unito.it (G.M.); 2 Istituto per lo Studio dei Materiali Nanostrutturati, Consiglio Nazionale delle Ricerche, via U. La Malfa 153, I-90146 Palermo, Italy; marialuisa.testa@cnr.it; 3 Acea Pinerolese Industriale S.p.A., Via Vigone 42, I-10064 Pinerolo (TO), Italy; roberta.gamberini@aceapinerolese.it; 4 NIS and INSTM Reference Centre, Via P. Giuria 7, I-10125 Torino, Italy

**Keywords:** orange, rhodamine, adsorption, photocatalysis, hybrid silica, wastewater treatment, waste-derived substances

## Abstract

Urban wastes are a potential source of environment contamination, especially when they are not properly disposed. Nowadays, researchers are finding innovative solutions for recycling and reusing wastes in order to favour a sustainable development from the viewpoint of circular economy. In this context, the lignin-like fraction of biomass derived from Green Compost is a cost-effective source of soluble Bio-Based Substances (BBS-GC), namely complex macromolecules/supramolecular aggregates characterized by adsorbing and photosensitizing properties. In this work BBS-GC were immobilized on a silica support (SBA-15) and the chemico-physical properties of the resulting hybrid material (BBS-SBA) were analysed by zeta-potential measurements, nitrogen adsorption at 77K and micro-calorimetric techniques. Successively, the BBS-SBA photosensitizing and adsorption abilities were tested. Adsorption in the dark of Rhodamine B and Orange II on BBS-SBA and their degradation upon irradiation under simulated solar light were shown, together with the formation of hydroxyl radicals detected by Electron Paramagnetic Resonance spectroscopy. Furthermore, the adsorption of six inorganic ions (Al, Ni, Mn, As, Hg, Cr) on BBS-SBA was studied in pure water at two different pH values and in a landfill leachate, showing the good potential of this kind of materials in the removal of wastewater contaminants.

## 1. Introduction

Nowadays, environmental preservation is one of the most important challenges for the scientific community. New technologies have been developed in order to avoid environmental damages and to promote depollution, that is, by limiting wastes accumulation and finding solutions for environment remediation. These technologies, when strongly interconnected, can contribute to a sustainable technological advancement and represent the frame of this work.

Although about 70 % of the Earth’s surface is covered by water, less than one percent can be classified as accessible freshwater and this shortage is expected to be further exacerbated by weather-related catastrophes, pollution levels and increased water consumption in populous countries such as China and India. Sustainable water management, infrastructures, access to a safe, reliable, affordable supply of water and adequate sanitation services improve living standards, expand local economies and lead to the creation of more job roles and greater social inclusion. From this point of view, wastewater treatments are fundamental steps for clean water supply after domestic/industrial use. The concern about the presence of highly polluting compounds produced in human activity (i.e., heavy metals, dyes, pesticides, herbicides, phenols, dioxins, Polycyclic Aromatic Hydrocarbons, nitrogen and phosphorous, etc.), together with new kinds of not commonly monitored refractory pollutants (Contaminants of Emerging Concern) detected at trace/sub-trace level in natural water bodies, are forcing scientific communities to find advanced strategies for depollution [1]. Beyond the traditional biological, physical and chemical depuration methods as biodegradation, adsorption, filtration, flocculation and ion exchange resins, other efficient oxidation technologies have been developed, having in common the generation of highly reactive species, in some cases induced by simulated or natural solar light (i.e., photocatalysis, ultrasounds/H_2_O_2_ or ozone; Fenton and photo-Fenton processes; cavitation oxidation) [2]. A particular attention has been paid to the exploitation and combination of traditional and innovative methods in tertiary water treatments, which provide a final depuration stage to raise the effluent quality to the desired level before water is reused, recycled or discharged into the environment.

On the other hand, population growth has causing the non-sustainable increase of municipal solid wastes, which are one of the most important by-products of the urban lifestyle, whose accumulation is even faster than urbanization rise. Global waste production is expected to increase to 2.2 billion tons per year [3]. Therefore, it is important to face this problem through a new and effective approach based on the principle of “closing the loop,” a perspective where all residues and wastes re-enter into production chain, creating economic, social and environmental added values [4]. 

Together with common recyclable materials, that is, glass, paper, metal, plastic, tires, textiles and electronics, also biodegradable wastes—such as food or garden mowing—can be recycled by composting, obviating to the problems of toxic leachates which derive from their disposal in landfill. In this paper the focus will be on the isolation and application in water remediation of macromolecular products derived from recycled green refuses. Indeed, in previous studies it has been demonstrated that this type of substances bears many similarities with humic acids, thus they can have a wide spectrum of applications, as fertilizers, surfactants, photosensitizers for pollutants removal, adsorbents for decontamination processes, as well as additive for preparation of fine chemicals [5]. Also, the exploration of their potential as metal ions adsorbents can be interesting, on the basis of literature studies on recycled materials such as cellulose nanofibers from medical cotton [6], sugarcane bagasse [7] and corn chaff [8] or by functionalized silica [9].

In a previous paper [10], some organic substances extracted from compost, namely CVT230, were immobilized on three different types of silica with peculiar porosity features and the resulting materials were successfully tested in the photodegradation of 4-methylphenol in aqueous solution. Among the different systems, the SBA-15 silica-based hybrid showed the best performances in terms of stability and reusability, which can be correlated to the silica morphology and texture, better allocating and stabilizing the waste-derived molecules. 

For these reasons, SBA-15 was used in the present study as support for an analogous type of Bio-Based Substances, obtained from Green Compost by a lab-scale procedure and labelled BBS-GC. BBS-GC and CVT230 were obtained by similar extraction procedures and their physical and chemical features are almost the same, as evidenced by a literature comparison. In both cases they can be considered supramolecular aggregates of molecules differing in molecular weight and constituted by aliphatic C-chains substituted by aromatic rings and containing several functional groups, such as COOH, CON, CO, PhOH and O-alkyl among others [11]. 

The hybrid material was characterized in terms of structure and surface properties and tested as adsorbent towards two differently charged dyes (the anionic Orange II and the zwitterionic Rhodamine B) and some inorganic ions: Al(III), Mn(II), Ni(II), Cr(III), Hg(II) and As(V). Furthermore, in order to promote the degradation of the adsorbed dyes, yielding also to a clean and re-usable material, the hybrid was exposed to simulated solar light irradiation, thus exploiting its photosensitizing features. Simultaneously, the generated reactive oxygen species (ROS) involved in the process were detected for the first time for this kind of hybrid.

## 2. Materials and Methods

### 2.1. Extraction of Bio-Based Substances-Green Compost (BBS-GC)

The bio-based substances (BBS-GC) were isolated from urban biowastes sampled from the Polo Ecologico process line of ACEA Pinerolese Industriale S.p.A. waste treatment plant in Pinerolo Italy. A green compost was obtained in the compost production section, from urban public park trimming and home gardening residues aged for more than 180 days. The isolation of BBS-GC was performed following a previously reported procedure [11] by treating green compost with NaOH 6 M water solution for 4 h at 60°C and separating the reaction mix by centrifugation. The liquid phase was then concentrated, different fractions were separated through a lab-scale ultrafiltration unit equipped with a membrane (molar mass cut-off 5 kDa) and the retentate fraction was then dried at 60 °C for 24 h and stored at room temperature. 

The chemical behaviour and the structure of the obtained compound are not significantly different from CVT230 used as functionalizing agent in a previous paper [10].

### 2.2. Synthesis of BBS-GC Immobilized on SBA-15

For the preparation of the hybrid BBS-GC/SiO_2_ sample a stepwise approach was used [10]. Firstly, the silica support SBA-15, was synthesized by using a non-ionic amphiphilic triblock polymer, Pluronic P123, as a template to control the porosity. Actually, this procedure was confirmed to induce a hexagonal ordered structure of the pores [12]. Then, the silica surface was functionalized by grafting aminopropyl groups through the reaction with (3-aminopropyl)triethoxysilane [13]. Afterwards, the covalent immobilization of BBS-GC on aminopropyl-silica (AMPS) was carried out by using a method [14] leading to covalent amide bond formation by the reaction of AMPS amino groups with the BBS-GC carboxylic groups activated by *N*-(3-dimethylamino propyl)–*N*′-ethylcarbodiimide hydrochloride (EDC). The obtained material was named BBS-SBA.

All the compounds used in the synthesis were purchased from commercial suppliers (Aldrich, Fluka, Milan, Italy) and used without further purification. Distilled toluene and dimethylformamide (DMF) were stored on molecular sieves (4 Å) to assure their complete dryness.

### 2.3. Characterization Techniques and Procedures

Water vapour uptake and molar heats of adsorption were evaluated following a previously reported procedure [15] by an adsorption microcalorimeter Tian-Calvet (Setaram, Lyon, France) equipped with a lab-made gas-volumetric apparatus for the quantitative study of surface-gas interactions. In the present work, samples were outgassed (<10^−3^ mbar) at 30 °C before adsorption experiments. Dosed amounts of steam were sent to the sample kept at 30 °C and left equilibrating. The heat released was recorded for each equilibrium steam pressure. The adsorption cycle was repeated after a 30 °C-outgassing of the sample under vacuum in order to evidence modifications induced by steam contact. 

ζ-potential measurements were performed on a Zetasizer (Malvern Instrument, Malvern, UK). The zeta potential values were measured using principles of laser Doppler velocimetry and phase analysis light scattering (M3-PALS technique). All the suspensions were prepared by dispersing 10 mg of powder in 20 mL of double distilled water.

Surface area and pore volumes were obtained by N_2_ adsorption at 77K in a ASAP2020 gas-volumetric apparatus (Micromeritics, Norcross, GA, USA). The samples were previously outgassed at 60 °C until a standard residual pressure of 10^−2^ mbar was present in the outgassing system. The specific surface area of BBS-SBA was calculated by the Brunauer–Emmett–Teller (BET) method [16], whereas the total pore volume and the pore size distribution were evaluated by applying the Density Functional Theory (DFT) model for cylinder pores [10].

The physico-chemical properties of BBS-SBA (reported in the Supplementary Materials section) are not so different from the material studied in the previous work (CVT230-SBA in Reference [10]), indicating a good reproducibility of the functionalization procedure.

### 2.4. Dyes Adsorption and Photodegradation

BBS-SBA (800 mg L^−1^) was used to adsorb 10 mg L^−1^ of dyes solutions (Orange II sodium salt, >85% and Rhodamine B, >95% purchased by Sigma Aldrich, Milan, Italy) at pH 5.5 which is the natural pH of the suspension. The BBS-SBA concentration was chosen as a result of a preliminary optimization procedure [10]. Before the experiment started, the suspension was stirred in dark conditions for 15 min in order to guarantee a good homogenization of the systems.

Photodegradation of both dyes was carried out with analogous homogenized suspensions and the trials were performed by irradiating the sample (5 mL) in closed Pyrex^®^ cells under continuous stirring. Solar radiation was simulated in a Solarbox (CO.FO.ME.GRA., Milan, Italy) equipped with Xenon lamp (1500 W) and cut-off filter for wavelengths below 340 nm (lamp irradiance 26 W m^−2^). At established times, after adsorption or irradiation, the samples were filtered through polytetrafluoroethylene (PTFE) membrane by using syringe Minisart^®^ and Syringe Filters 0.45 μm. In order to evaluate the abatement due to either photodegradation or adsorption phenomena, the dyes were desorbed by adding 20 mL of acetonitrile after the photodegradation trials. The resulting suspension was left under stirring for 6 hours in the dark, then filtered and analysed by High Performance Liquid Chromatography (HPLC) as described below.

The effect of the bare SBA-15 silica support was also evaluated in the adsorption of both dyes, in the same experimental conditions adopted during trials carried out with BBS-SBA.

In order to verify the possible contribution of detached BBS-GC in homogeneous phase, a suspension of 1600 mg L^−1^ of BBS-SBA in MilliQ™ water was stirred for 30 min during light exposition. Then the powder was filtered and the photosensitizing activity of the liquid phase (labelled as filtered BBS-SBA water, F-BS-W) was tested towards Orange II: 2.5 mL of F-BS-W and 2.5 mL of Orange II 20 mg L^−1^ were put in the cell and irradiated as described above. 

The dyes photobleaching was monitored by a double-beam UV-visible spectrophotometer CARY 100 SCAN (Varian, Palo Alto, CA, USA). A sample quartz cell of 1 cm path length was used. The measurements were performed at the maximum absorbance of the dyes (485 nm for Orange II and 555 nm for Rhodamine B). 

Degradation of dyes was followed by High Performance Liquid Chromatography (HPLC), employing a Merck-Hitachi instrument equipped with Lichrospher RP-C18 column (125mm × 4 mm i.d., d.p. 5 mm, from Merck, Darmstadt, Germany), Rheodyne injector, L-6200 pumps, UV-Vis Merck Hitachi L-4200 detector and D-7000 HPLC System Manager (HSM) software (Darmstadt, Germany). Orange II and Rhodamine B were eluted under isocratic condition with acetonitrile/ammonium acetate 5mM solution (75/25) for Orange II and water/methanol (25/75) for Rhodamine. The flow rate was 1.0 mL min^−1^ and the retention time was about 6 minutes in both cases. The detector was set at wavelength 485 nm and 555 nm in order to reveal Orange II and Rhodamine, respectively. In the case of Orange II, a brief column cleaning with 100% acetonitrile was performed every three samples analysis.

The study of the reactive species generated during photocatalytic experiments was conducted by Electron Paramagnetic Resonance (EPR) in the presence of 5,5-Dimethyl-1-pyrroline-N-oxide (DMPO) and 2,2,6,6-tetramethyl-4-piperidone hydrochloride (4-oxo-TMP) as spin-traps.

EPR spectra were recorded at room temperature with a ESR 300E X-band spectrometer (Bruker Italia s.r.l., Milan, Italy). The acquisition parameters were as follows: frequency = 9.783 GHz, microwave power = 5 mW, centre field = 3470 G, sweep width = 80 G, receiver gain = 1 × 10^5^, modulation amplitude = 0.41 G, conversion time = 40.96 ms. The experiments were carried out by adding the spin trap in the cell after 2 hours irradiation; after 15 minutes of incubation in the same experimental conditions, a portion of sample was withdrawn in a capillary quartz tube and the EPR spectrum was immediately acquired.

### 2.5. Inorganic Ions Adsorption

Aqueous solutions of Aluminium, Nickel, Manganese, Chromium, Mercury and Arsenic, prepared by concentrated commercial standards Tritisol^®^ in MilliQ^®^ water (Al(NO_3_)_3_·9H_2_O, NiCl_2_, MnCl_2_, Hg(NO_3_)_2_, CrCl_3_, As_2_O_5_), were put in contact at 25°C with BBS-SBA in a beaker and left under mechanical stirring at 500 rpm throughout the measurement. During the experiment, pH and temperature were continuously monitored by means of a pH electrode and a thermometer introduced in the beaker. At different times, 10 mL of suspension were withdrawn and filtered with a cellulose filter (0.45 μm, Minisart, Sartorius, Göttingen, Germany) supported on syringes with plungers devoid of rubbery parts (BD Discardit^TM^) to remove powdery adsorbent. After filtration, 10 μL of ultrapure HNO_3_ (65%, Suprapur^®^, Merck) were added to each sample and the solutions were stored at 4 °C until further analysis. Ions concentration was determined by Inductively Coupled Plasma Optical Emission Spectrometry (ICP-OES), model Optima 7000 DV (Perkin Elmer, Waltham, MA, USA), equipped with a cross-flow nebulizer, a Scott spray chamber and a double monochromator (prism and Echelle grating). The instrumental conditions were: plasma power 1.3 kW, sample aspiration rate 1.5 mL min^−1^, argon nebulizer flow 0.8 L min^−1^, argon auxiliary flow 0.2 L min^−1^ and argon plasma flow 15 L min^−1^.

Preliminary tests were carried out by adding BBS-SBA (800 mg L^−1^) in solutions of a single inorganic ion (1 × 10^−4^ M). The pH was modulated by adding NaOH or HNO_3_ solutions (0.2 M) in order to reach the stable pH values of 3 and 5, chosen to avoid precipitation problems. The experiment duration was one hour, sufficient to evaluate the whole adsorption process. The effect of the bare SBA-15 silica support was also evaluated in adsorption tests towards Nickel, in the same experimental condition used to test BBS-SBA.

Moreover, the hybrid adsorption properties were investigated in presence of: (i) a solution containing all the species (1·× 10^−5^ M for each ion) at pH 5; and (ii) a landfill leachate from Acea Pinerolese S.p.A. tested without any modification (pH 5.6). In both cases, the adsorption process was slower than the preliminary tests, therefore the adsorbed amounts were evaluated after 6 hours of mixing.

Given the origin of BBS-GC from residual biomass and their extremely heterogeneous composition, the release of metals from the hybrid was estimated by suspending it in MilliQ^®^ water (800 mg L^−1^) in order to detect eventual interferences during adsorption experiments.

## 3. Results

### 3.1. BBS-SBA Structural Properties

The structural characterization procedures were described elsewhere [10] and the main results were reported in the Supplementary Material section. As shown by the Transmission Electron Microscopy (TEM) image (Figure S1) and from Thermogravimetric analysis, TGA (Figure S2), the results obtained using BBS-GC as functionalising agent are not significantly different from those obtained using CVT230.

### 3.2. BBS-SBA Surface Properties

**Figure 1:**
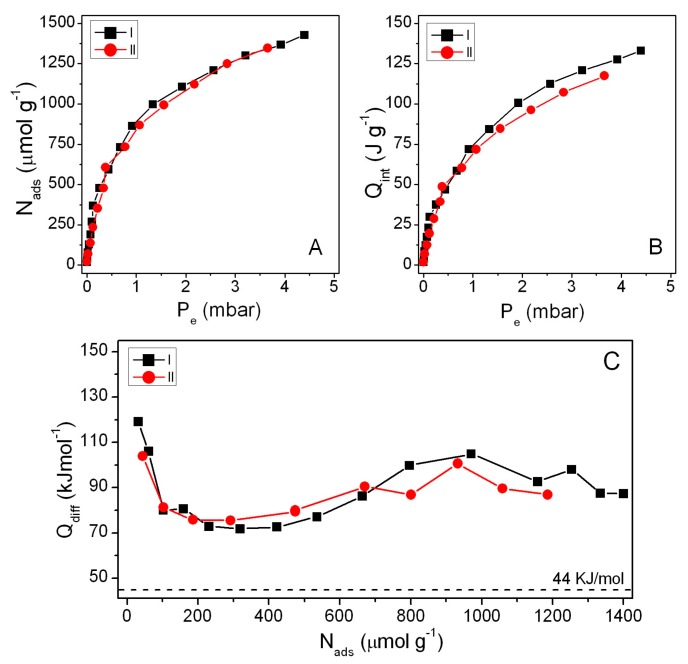
Water-adsorption data for two cycles: (**a**) adsorption isotherms, (**b**) calorimetric curves, (**c**) differential heat of adsorption.

BBS-SBA surface features were studied by water steam adsorption and ζ-potential measurements at room temperature and by N_2_-adsorption at 77 K. In Figure 1, data obtained at 298 K by means of an adsorption microcalorimeter equipped with a gas-volumetric apparatus on sample outgassed *in vacuum* at the same temperature are reported. 

The water vapor pressure in the sample cell was progressively increased by sending known amounts of vapor. Thus, as shown in Figure 1a,b, the amount of water adsorbed (*N_ads_*) and the heat developed by water adsorption (*Q_int_*) were plotted as function of the water vapor pressure at the equilibrium (*P_e_*). The differential heat of adsorption *Q_diff_*, Figure 1c, represents the molar adsorption heat evolved for each dose of water adsorbed on the sample surface and is used to determine the energy as a function of surface coverage. It was calculated from the data in Figure 1a,b by the following Equation (1):

(1) $$Q_{diff}=\frac{\Delta Q_{int}}{\Delta N_{ads}}$$

Two cycles of adsorption/desorption were performed in order to assess the reversibility of the process and the eventual differences after consecutive surface coverage. Actually, Figure 1a,b show as the two adsorption cycles are approximately equivalent, both in terms of amount of water adsorbed and evolved heat, demonstrating that the steam contact does not modify the surface of the sample. Moreover, experimental data also show that desorption is complete, thus allowing the surface sites to be further available after a simple outgas at 30 °C. 

On the other hand, the values of differential heat of adsorption, reported in Figure 1c as a function of coverage, demonstrate that the interaction between the surface of BBS-SBA and water steam is extremely favoured and energetic. In the range of pressures examined, the heats evolved present some fluctuation, probably because water molecules allow some rearrangement of BBS-GC molecules at the SBA surface. All the data were significantly higher than 44 kJ mol^−1^, namely the liquefaction molar enthalpy of water vapour, which can be taken as threshold reference value to classify a surface as hydrophilic or hydrophobic, indicating the surface as highly hydrophilic and able, at least in principle, to interact with polar substrates. 

**Figure 2:**
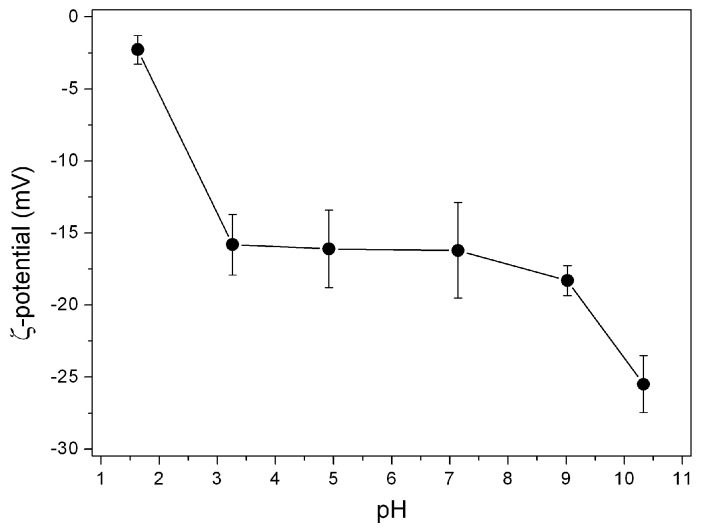
ζ-potential values for BBS-SBA at different pHs.

The high hydrophilicity obtained by microcalorimetry analysis suggests the presence of charged groups at the BBS-SBA surface. This hypothesis is confirmed by the results of the ζ-potential determination at different pH values showed in Figure 2. The strongly negative surface evidenced by this analysis is attributable to two main factors: the presence of many dissociable carboxylic groups in the BBS-GC molecules [11] and the typical negative charge of silica support [17,18]. The N_2_-adsorption measurements at 77 K (Figure S3) permit to obtain useful information on the modification induced on the SBA surface by BBS-GC coating. In agreement with previous data concerning the grafting of CVT230 on SBA-15, the surface area was dramatically reduced from 845 to 70 m^2^ g^−1^, as well as the pores dimension and volume decrease from 100 to 80 Å and from 2.5 to 0.5 cm^3^ g^−1^, respectively. 

From these data, it is evident that BBS-GC molecules partially occlude the SBA-15 pores, introducing severe modifications on the surface characteristics of the final system but adding new properties due to the BBS-GC functional groups. In order to identify these new behaviours, BBS-SBA was employed in the treatment of different aqueous systems.

### 3.3. BBS-SBA Behaviour in the Treatment of Organic Dyes Solutions

**Figure 3:**
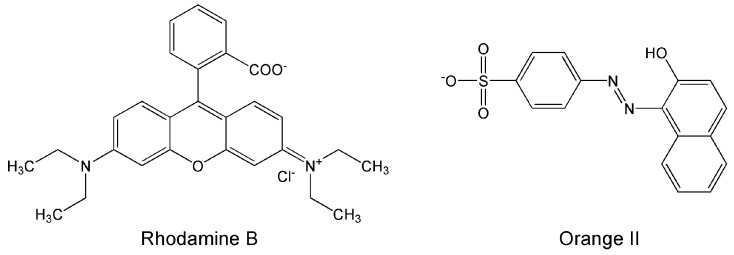
Chemical structures of Orange II and Rhodamine B at pH 7.

On the basis of the surface characterization described above and the photosensitizing properties of BBS-GC, the interactions between BBS-SBA and the organic dyes Orange II and Rhodamine B (their chemical structures are shown in Figure 3) were studied both in the dark and under irradiation with simulated solar light, as described in Section 2.4. Parallel experiments were also carried out in order to understand the relative contribution of adsorption and photodegradation phenomena. 

These two dyes were selected since they are used as reference models in many studies concerning the photocatalytic properties of different materials [19,20,21,22], Moreover, they are differently charged at pH 5.5: Orange II is negatively charged [23], whereas Rhodamine B presents its zwitterionic form [24].

**Figure 4:**
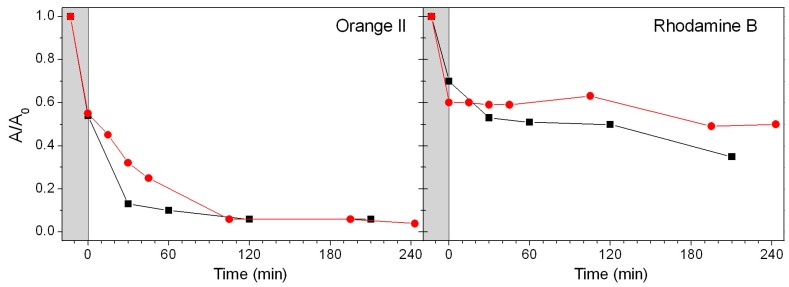
Bleaching of Orange II and Rhodamine B water solutions (10 mg L^−1^) *vs.* time in the presence of BBS-SBA (800 mg L^−1^) in the dark (red circles) and under irradiation with simulated solar light (black squares). The grey area represents the initial step (15 min in the dark) described in Section 2.4.

As described in the Section 2.4, a water suspension containing BBS-SBA and Orange II or Rhodamine B was initially stirred in the dark for 15 minutes in order to homogenize the system. Then, the mixture was irradiated by simulated solar light or left in the dark, for almost 4h and the bleaching of Orange II or Rhodamine B solutions at different times was determined by UV-visible spectroscopy. The experimental data (Figure 4) highlight an immediate interaction between both the dyes and the BBS-SBA surface, with disappearance of 40–45 % of each dye from the solution during the preliminary homogenization. 

On the other hand, the successive behaviour is different for the two dyes. In the case of Orange II, the abatement is almost complete after 4h for both tests but the removal under irradiation is faster, reaching about 90% in 30 minutes. On the contrary, the experiments with Rhodamine B evidence as the removal in the dark is approximately limited to the 40–50 % observed in the initial step of the experiment, whereas under irradiation the concentration of Rhodamine B in the reaction system slowly decreases throughout the experiment. In order to confirm these results, all the samples obtained during the experiments were also analysed by HPLC. In the case of Orange II experiments, the HPLC data are in substantial agreement with the UV-visible results, whereas, in the case of the Rhodamine B solution under irradiation, a difference was recorded, indicating the formation of coloured intermediates absorbing in the same wavelengths range. 

It is worth to consider that the decrement in dyes concentration in the dark is due to their adsorption on the BBS-SBA surface, whereas it has to be assessed if the colour disappearance under irradiation is ascribable to both adsorption and photodegradation mechanisms. 

First of all, the influence of eventually detached BBS-GC from the support, acting as homogeneous photosensitizer, was investigated. A suspension of BBS-SBA in water was irradiated under stirring for 30 min. Then the powder was filtered and the filtrate (F-BS-W) was added to an Orange II solution and irradiated for 4h. In these conditions, no dye degradation occurred, confirming that the detached portion of BBS-GC is negligible and all the events take place thanks to the activity of the heterogeneous photosensitizer. 

In order to distinguish the adsorption phenomenon concurrently with the photodegradation, a desorption procedure was carried out with acetonitrile at the end of the irradiation. The global amount of desorbed dye was 50% for Orange II, indicating that the effectively reacted (photo-degraded) portion of the dye was about 45%. On the other hand, in the case of Rhodamine B, the BBS-SBA photodegradation effectiveness can be estimated in about 30% of the initial amount of dye. 

These data suggest a combined effect adsorption/photodegradation in the dye removal. For what concerns the adsorption phenomenon, the strong adsorption of Orange II on BBS-SBA is not sustainable on the basis of an electrostatic attraction. They are both negatively charged in the experimental conditions, therefore some physical adsorption mechanism should be invoked. Since analogous experiments, carried out on the non-modified SBA-15, did not give any dye adsorption, the adsorption properties of BBS-SBA should be completely attributable to the BBS-GC composition and are probably the result of the presence of both hydrophilic functional groups and hydrophobic chain on the BBS-SBA surface. On the other hand, the interaction between Rhodamine B and BBS-SBA surface seems to be rather less important than in the case of Orange II, although the presence of a positive charge should favour the interaction with BBS negatively charged functional groups. Indeed, it is important to recall that the morphology of the mesoporous SBA-15 system is drastically changed by BBS functionalization, which is responsible of an important decrease of specific surface area and porosity of the material probably caused by pore obstruction. The Rhodamine B molecule is significantly larger than Orange II and probably the steric hindrance blocks the molecule at material external surface avoiding the access to the pores internal surface, consequently limiting its adsorption. 

The material after irradiation is expected to remain unaltered in the structural behaviour but probably slightly modified in the surface properties, as reported in Reference [10]. In that case the material functionalised with CVT230 (analogous in chemical and structural behaviours with respect to BBS-GC used in this work) was submitted to a prolonged irradiation of up to 45 hours and the treatment caused only a limited degradation of the functionalising agent, corresponding to about 1% of the total weight (measured by TGA). Moreover, a slight increase in the surface area of the material was observed, suggesting a partial degradation of the organic molecules.

To study the mechanism involved in photodegradation process, the presence of radical species during the degradation of Orange II and Rhodamine B was studied by EPR spectroscopy, since the photodegradation of dyes in water solution is usually related to the formation of reactive oxygen species (ROS) [25]. Following a previously reported procedure [26,27], a sample of BBS-SBA was irradiated under simulated solar light for 2h, then DMPO or 4-oxo-TMP spin-traps were added. The resulting mixture was irradiated again for 15 min and the related EPR spectrum was collected immediately after irradiation. 

**Figure 5:**
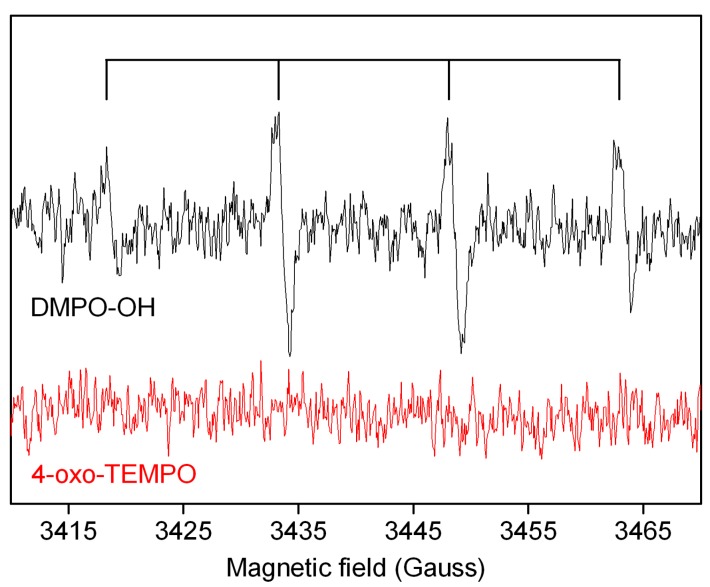
EPR spectra obtained by irradiation of BBS-SBA in the presence of DMPO and 4-oxo-TMP. The lines on the top highlight the typical 1:2:2:1 pattern of the DMPO-OH adduct.

As can be seen in Figure 5, in the presence of DMPO the typical spectra of the DMPO-OH adduct was obtained after irradiation, indicating the generation of hydroxyl radicals by BBS-SBA, whereas no signal of the 4-oxo-TEMPO adduct, due to the presence of singlet oxygen, has been recorded. Moreover, also the spectrum ascribable to superoxide radical adduct with DMPO [28] cannot be revealed in these experimental conditions. The preferential generation of hydroxyl radicals can be the discriminating factor for the higher levels of photodegradation occurred in presence of Orange II than Rhodamine B [19], since the Rhodamine B degradation seems to be mainly dependent to the presence of O_2_^−^• radicals [29] rather than to the presence of OH• [30]. The reaction mechanism should pass through the study of reaction intermediates and will be carried out in a future work.

### 3.4. Adsorption of Inorganic Ions on BBS-SBA

The adsorption properties of SBA-BBS towards six inorganic ions, Al(III), Ni(II), Mn(II), Cr(III), Hg(II) and As(V), were studied at 25 °C in different experimental conditions. The entity of adsorption was determined by ICP-OES after filtration of a sample of the reaction mixture, withdrawn at the desired reaction time and successively acidified with HNO_3_, in order to eliminate the BBS-SBA powder and avoid precipitation of salts or hydroxides of the ions. 

**Figure 6:**
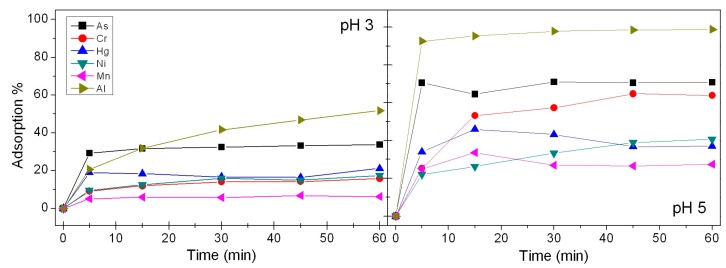
Adsorption of Al(III), Ni(II), Mn(II), Cr(III), Hg(II) and As(V) solutions 1 × 10^−4^ M at pH 3 (**left**) and pH 5 (**right**) on BBS-SBA (800 mg L^−1^).

Preliminary tests were done at pH 3 and 5 with single-ion 1 × 10^−4^ M solutions (Figure 6). The adsorption of each species was followed for 1 hour but the adsorption process seems to be almost concluded in very short times (5–10 min), with the only exception of Al(III) at pH 3. Comparing the results at different pH, it is clear that the adsorbed amount is higher at pH 5 for all the species. Even if the ζ-potential values measured at the two pH values are not showing relevant differences (Figure 2), the effect of higher dissociation degree of carboxylates on the BBS-SBA surface probably allows a more efficient chelating effect at the base of the interaction mechanism towards the studied substrate.

Moving to the analysis of the behaviour of the different ions: Al(III) reaches a 99% removal in few minutes at pH 5, its adsorption on a negatively charged surface is clearly favoured by its small dimensions and high positive charge; Cr(III), Ni(II) and Mn(II) adsorption doubles at pH 5, allowing a good removal from the solution, whereas 40% of Hg(II) adsorption at the same pH is an interesting result, considering the toxicity of this species; As(V) is mainly present as oxyanion at this pH [31] but it is quickly removed up to 70% at pH 5, probably due to a physical adsorption on the material.

Similar tests performed on the bare SBA-15 silica showed that its adsorptive features toward inorganic ions are negligible. In agreement with the results obtained with the two dyes, the predominant role of BBS-GC in the interaction with substrates was highlighted. 

Moreover, due to the heterogeneous nature of BBS-GC (that include a consistent inorganic portion), a possible intrinsic ions release by BBS-SBA (800 mg L^−1^) in ultrapure water was also investigated. ICP-OES analysis of the resulting solution did not reveal a significant presence of ions, except for a very low amount of Al(III) (50 ng L^−1^), showing that the material performances can be considered net of impurities interferences. 

Then, the material was studied towards the removal of the same ions mixed together at pH 5, the best conditions in term of adsorption, to simulate a real wastewater treatment. In order to optimize the contact time between the material and the multiple-ions solution, the experiment was carried out up to 6 hours. The results (reported in Table 1) demonstrated that the adsorption process is slower with respect to the experiments conducted on single-ion solutions. Despite the lack of As(V) and Mn(II) removal, the increased matrix complexity did not affect the adsorption of the other four ions. Moreover, preliminary results obtained after longer time of contact (data not shown), showed that the ions sequestration further increased with time.

**Figure 7:**
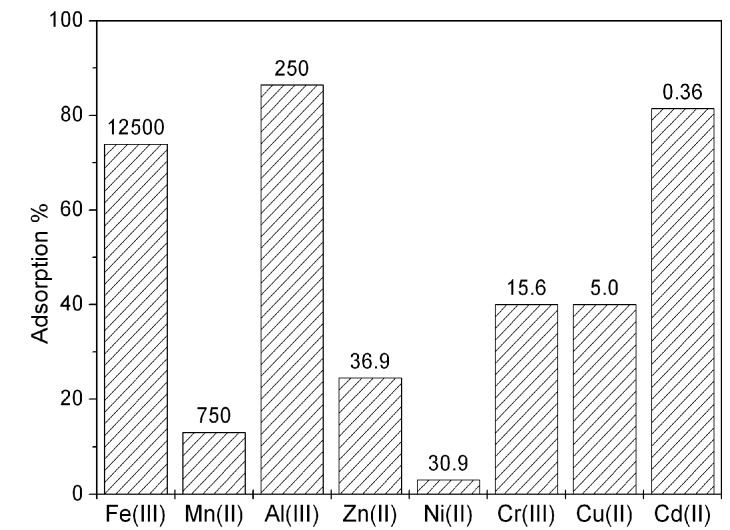
Results of the treatment of landfill leachate with BBS-SBA at pH 5.6 for 6 hours. The labels indicate the starting concentration of each ion (ng L^−1^).

On the basis of these good results, a final test was made on a real landfill leachate containing variable amount of metal ions after 6 hours of contact time (Figure 7). The results are encouraging, since also in these conditions the BBS-SBA material maintains its ability to remove the metal ions previously studied, together with a good affinity for other toxic species like Cu(II) and Cd(II).

## 4. Conclusions

The BBS-SBA hybrid is a stable, well characterized material with interesting properties in the removal of different species from aqueous solutions. The hydrophilic surface, due to the presence of several dissociable groups on the BBS-GC precursor used for the synthesis, allowed a good interaction with charged substances, particularly evident in the experiments in presence of inorganic ions. Although detailed studies about the role of pH, temperature and dosage are needed to determine the adsorption properties of BBS-SBA, preliminary tests carried out with a real landfill leachate are encouraging since underline the possible use of this material also in complex matrices and with variable concentrations of analytes. 

On the other hand, the hydrophobic moieties of the complex structure of BBS-GC play a determining role in the strength of interaction with organic molecules, as showed in the case of Orange II and Rhodamine B adsorption. The production of hydroxyl radicals upon BBS-SBA irradiation foreshadows a possible higher efficiency in organic pollutants removal, due to the synergy between adsorption and photocatalytic degradation.

## Figures and Tables

**Table 1:** Adsorption % of Al(III), Ni(II), Mn(II), Cr(III), Hg(II) and As(V) (1 × 10^−5^ M each) mixed together in the presence of BBS-SBA (800 mg L^−1^) at pH 5.

Ion	Adsorption % after 6 h
As(V)	0
Cr(III)	42
Hg(II)	82
Ni(II)	64
Mn(II)	0
Al(III)	31

## Supplementary Materials

The following are available online at http://www.mdpi.com/2079-4991/9/2/162/s1, Figure S1: TEM image of BBS-SBA material, Figure S2: TGA curves of SBA and BBS-SBA materials obtained in air with a temperature ramp of 10 °C min^−1^, Figure S3: N_2_ adsorption–desorption isotherms at 77 K and mesopore size distribution of SBA and BBS-SBA after thermal activation *in vacuo* at 60 °C.
