# Supplementary material for: Green Waste-Derived Substances Immobilized on SBA-15 Silica: Surface Properties, Adsorbing and Photosensitizing Activities towards Organic and Inorganic Substrates
Source: Nanomaterials (Basel). 2019 Jan 29;9(2):162. doi: 10.3390/nano9020162 (PMC6409592; doi:10.3390/nano9020162)
Supplement: Supplementary file 1 [file nanomaterials-09-00162-s001.pdf]

nanomaterials

Supplementary materials

Green Waste-Derived Substances Immobilized on SBA-15 Silica: Surface Properties, Adsorbing and Photosensitizing Activities towards Organic and Inorganic Substrates

Maria Laura Tummino[1], Maria Luisa Testa[2], Mery Malandrino[1], Roberta Gamberini[3], Alessandra Bianco Prevot[1], Giuliana Magnacca[1,4], Enzo Laurenti[1,*]

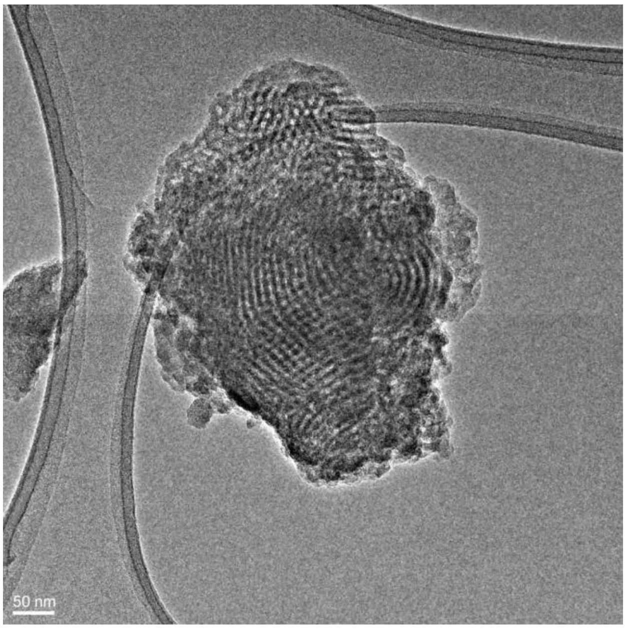

Figure S1. TEM image of BBS-SBA material

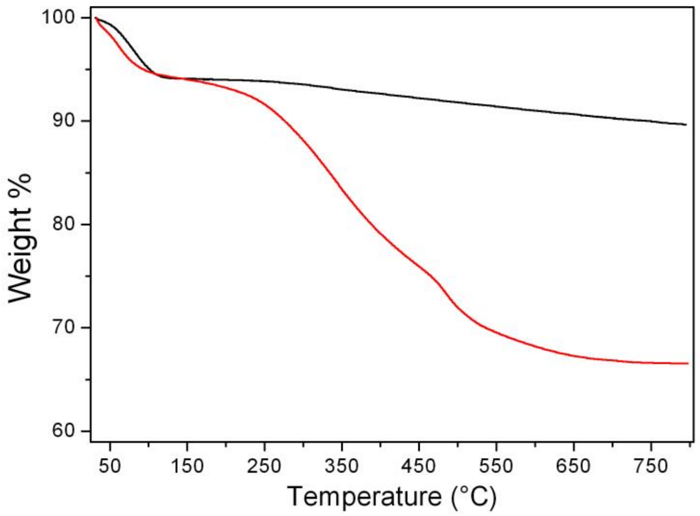

Figure 2. TGA curves of SBA (black line) and BBS-SBA (red line) materials obtained in air with a temperature ramp of 10°C min^{-1}. The amount of organics, determined as difference between the weight loss at 800°C of BBS-SBA and SBA materials, corresponds to 23%, comprehensive of linker and BBS. Further details in ref [10].

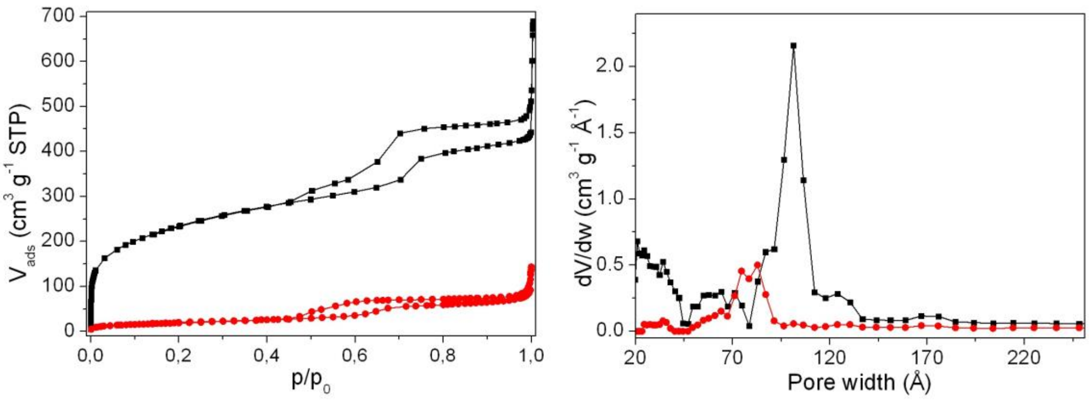

Figure 3. N_2 adsorption–desorption isotherms at 77 K (left) and mesopore size distribution (right) of SBA (black) and BBS-SBA (red) after thermal activation *in vacuo* at 60°C.
